# Feasibility of Low-Dose Contrast Medium High Pitch CT Angiography for the Combined Evaluation of Coronary, Head and Neck Arteries

**DOI:** 10.1371/journal.pone.0090268

**Published:** 2014-03-03

**Authors:** Zhiwei Wang, Yu Chen, Yining Wang, Huadan Xue, Zhengyu Jin, Lingyan Kong, Jian Cao, Shuo Li

**Affiliations:** Department of Radiology, Peking Union Medical College Hospital, Beijing, China; Northwestern University Feinberg School of Medicine, United States of America

## Abstract

**Purpose:**

To evaluate the image quality and radiation dose of combined heart, head, and neck CT angiography (CTA) using prospectively electrocardiography (ECG)-triggered high-pitch spiral scan protocol, compared with single coronary CTA.

**Materials and Methods:**

151 consecutive patients were prospectively included and randomly divided into three groups. Group 1 (n = 47) underwent combined heart, neck, and head CTA using prospectively ECG-triggered high-pitch spiral (Flash) scan protocol with a single-phase intravenous injection of iodinated contrast and saline flush; Group 2 (n = 51) underwent single coronary CTA with Flash scan protocol; and Group 3 (n = 53) underwent single coronary CTA with prospective sequence scan protocol. All patients were examined on a dual source CT (Definition FLASH). The image quality was determined for each CT study.

**Results:**

Patients of scanning protocol Group 1, 2, and 3 showed no significant differences in age, sex, heart rates, and BMI. Evaluation of coronary artery image quality showed comparable results in the three scanning protocol groups on a per patient-based analysis. In group 1, image quality was found to be sufficient to be diagnostic in all arterial segments of carotid arteries. The mean dose-length product (DLP) for group 1 was 256.3±24.5 mGy×cm and was significantly higher in comparison with group 2 (93.4±19.9 mGy×cm; p < 0.001). However, there was no significant difference of DLP between group 1 and group 3 (254.1±69.9 mGy×cm).

**Conclusions:**

The combined heart, neck, and head arteries scan using prospectively electrocardiography (ECG)-triggered high-pitch spiral scan protocol in 1 single examination resulted in an excellent opacification of the aorta, the carotid arteries, and the coronary arteries and provided a good image quality with low radiation dose.

## Introduction

Cardio-cerebral vascular disease is the leading cause of mortality worldwide [1]–[2]. Atherosclerosis is a common pathogenesis, a diffuse process that affects multiple vessels including the carotid and coronary arteries [3]. The coexistence rate of these two diseases has been reported at multiple imaging modalities, including digital subtraction angiography (DSA), Doppler ultrasonography (US), and multi-detector CT angiography (CTA) [3]–[6]. CTA has emerged as a reliable tool in the evaluation of atherosclerotic disease and has been shown to have a high concordance with histology when characterizing carotid artery plaque [7]–[8]. Additionally, CTA has evolved into the modality of choice for anatomic evaluation of the coronary arteries with high sensitivity for the detection of significant coronary artery stenosis [9]–[14]. However, the benefits of CTA must be weighed against the potential risks associated with radiation exposure. In retrospective electrocardiogram (ECG)-gated spiral acquisition mode, radiation doses up to 12 mSv have been reported for coronary CTA (cCTA) [15]. If a combined coronary, head, and neck CTA examination is undergone with this protocol, an even higher cumulative radiation dose would be expected. An additional limitation of CTA is the necessary administration of iodinated contrast media, which may contribute to renal impairment [16], [17]. With a multiple-target, multiple-acquisition examination, the total contrast media dose will be increased significantly.

The second generation of the dual-source 128-slice CT system (Definition Flash, Siemens Healthcare, Forcheim, Germany) provides a wider coverage of 38.4 mm; this, along with faster gantry rotation, have improved temporal resolution to 75 milliseconds, allowing spiral acquisitions to be performed at a pitch as high as 3.4 [18], [19]. A prospectively ECG-triggered high-pitch spiral (Flash mode) scan protocol allows a cCTA to be performed within 270 ms while keeping the effective radiation dose below 1 mSv [20]–[24]. The high-pitch Flash mode provides gapless data from the dual-source CT system, reducing motion artifacts over the large scan range. We propose that with a high-pitch protocol, it may be feasible to perform a combined carotid and coronary CTA within a single helical acquisition, utilizing a single contrast media bolus.

The purpose of this randomized study is to evaluate the image quality and radiation dose of a combined heart, neck, and head CTA using a high-pitch scan protocol with a low contrast media dose in comparison with both coronary-only high-pitch cCTA and prospective ECG-trigger cCTA.

## Materials and Methods

This study was approved by Peking Union Medical College Hospital ethics committee. Written informed consent was obtained from all patients. Between June 2012 and September 2012, 219 patients were clinically referred for cCTA at our institution. Patients who had a history of percutaneous intervention (n = 27) or bypass surgery (n = 11) were not eligible for our study and were excluded. Patients with known allergy to iodinated contrast material (n = 5), impaired renal function (serum creatinine level, >120 μmol/L; n = 9), or persistent arrhythmias (n = 11) were excluded. Thus, 156 consecutive patients were eligible for inclusion in this study.

**Figure 1:**
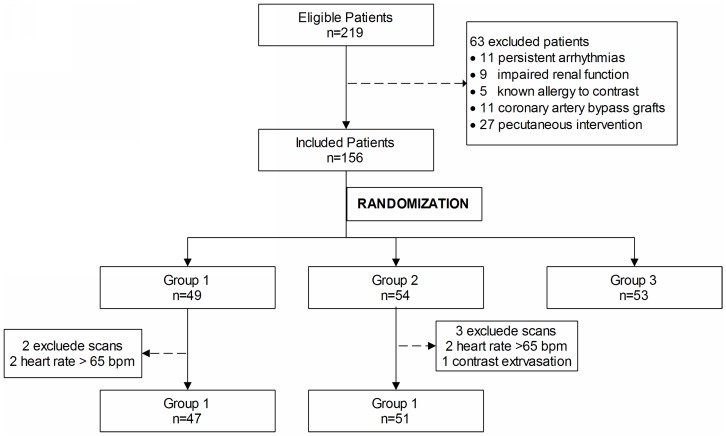
Process of the patients’ enrollment.

Study patients were randomly divided into three groups. Patients in Group 1 (n = 49) received a combined heart, neck, and head prospective ECG-triggered high-pitch CTA; Group 2 (n = 54) underwent high-pitch cCTA, and Group 3 (n = 53) underwent prospective ECG-triggered sequence scan cCTA. All patients received nitroglycerin (0.4 mg per dose) sublingually just prior to the CT scan. All patients with a heart rate above 65 beats per minute, in the absence of contraindications, received a single dose of 100 mg metoprolol (AstraZeneca, China) 1 hour prior to scanning. A heart rate greater than 65 beats per minute with contraindications to β-blockers or patients not properly responding to the administered β-blocker were excluded. Finally, 151 patients were eligible for analysis (Fig 1).

### CT Scan Protocols and Data Reconstruction

All CTs was performed using a 128-slice DSCT system (Somatom Definition Flash, Siemens Healthcare, Forchheim, Germany). Detector collimation is 2×64×0.6 mm. A z-axis flying focal spot is applied with an acquisition of 2×128 slices per rotation. Both tubes were operated at 120 kV. Scout-based automatic tube current modulation (CareDose 4D, Siemens healthcare, Forcheim, Germany) was used with the reference tube current–time product set at 320 mAs per rotation. The pitch was 3.2 for group 1, and 3.4 for group 2. For all protocols, patients were positioned supine on the CT table with both arms along the chest. Contrast media (Ultravist 370 mgI/ml, Bayer Schering) and saline chaser were administered at 5 mL/s using a dual-head power injector (Empower, ACIST) into an antecubital vein through an 18-gauge catheter. Heart rate and ECG trace were recorded during examinations.

#### Protocol for Group 1

A test bolus scan was performed to determine the transit time. An injection of 15 mL of iodinated contrast media was followed by a 30 mL saline chaser. The time until the peak opacification in the proximal ascending aorta was measured and this time added 3 seconds was considered to represent the transit time of contrast agent. Sixty-five mL of contrast agent were injected, followed by a 50 mL saline chaser. The scan was initiated with a delay according to the transit time. The scan was performed in a caudocranial direction, starting above the diaphragm, just below all cardiac structures, and ending at the apex of the skull.

#### Protocol for Groups 2 and 3

Fifty-five milliliters of contrast media followed by a 50 mL saline chaser were administered. with bolus tracking using a region of interest (ROI) in the ascending aorta. The scan was automatically triggered when the tracking ROI reached a threshold of 100 Hounsfield units (HU) above baseline attenuation. Scan direction was craniocaudal, starting above the coronary ostia and ending at the diaphragm below all cardiac structures. In the high-pitch spiral mode (Group 2), prospective ECG-triggering was used to obtain a complete dataset in a single heart beat [20] starting at 55% of the R–R interval. In sequential mode (Group 3), The centre of data acquisition window was set at 70% of the R-R interval[21]. The entire heart was covered in three or four heart beats in a step-and-shoot fashion.

### Image Reconstruction

Axial sections for the entire dataset (1.0 mm, increment 0.6 mm) were reconstructed using a medium-soft convolution kernel (B26). Datasets for coronary arteries were reconstructed with a slice thickness of 0.6 mm, an increment of 0.4 mm, a field of view of 180 mm, a medium-soft convolution kernel (B26) and additionally a sharp convolution kernel (B46) in patients exhibiting coronary calcium.

### Image Evaluation

All reconstructed images were transferred to a dedicated workstation (MMWP, Siemens Healthcare, Forchheim, Germany). Axial images, multiplanar reformations, and maximum intensity projections were used to evaluate arteries.

Coronary artery segments were classified according to a modified American Heart Association 17-segment model [25]. Segments were evaluated if luminal diameter met or exceeded 1.5 mm, as judged by two independent observers (each with more than 5 years of CT coronary angiographic experience). For any disagreement in data analysis, a consensus reading was performed. Image quality was semi-quantitatively assessed using a four-point grading scale: (1) excellent (no artifacts, unrestricted evaluation), (2) good (minor artifacts, good diagnostic quality), (3) adequate (moderate artifacts, still acceptable and diagnostic), and (4) not assessable (severe artifacts impairing accurate evaluation). Images with a score of 1–3 were considered acceptable for diagnosis [26].

Subjective image quality was also evaluated by two independent readers (each with more than 5 years of CT head and neck angiographic experience) for carotid arteries using a four-point scale [27]: (1) excellent: excellent image quality enabling sufficient differentiation of even small structures; (2) good quality: good image quality providing all details necessary for an adequate diagnosis; (3) poor-diagnostic: patency or occlusion of the vascular segment can be assessed, but image quality is unsatisfactory; (4) non-diagnostic: vascular segments cannot be assessed because of streak artefacts and graininess of the images. And we used the middle cerebral artery (MCA) branches as an indicator for evaluation of intracranial vessels using a four-point grading system as follows [28]: grade 1, two or more M2 branches demonstrated; grade 2, only one M2 branch demonstrated; grade 3, complete demonstration of M1 but no demonstration of M2; and grade 4, incomplete demonstration of M1. For any disagreement in data analysis, a consensus reading was performed.

Objective evaluation of image quality of the coronary arteries was measured as image noise, attenuation (measured in Hounsfield Units, HU), and contrast of the coronary lumen, as well as signal-to-noise ratio (SNR) and contrast-to-noise ratio (CNR) [25], [26], [29]. Image noise was defined as the standard deviation of attenuation in a ROI placed immediately cranial to the left coronary ostium in the aortic root. The ROI was chosen to be as large as possible while carefully avoiding inclusion of the aortic wall to prevent partial volume effects. Attenuation within the lumen of the coronary arteries was measured by placing the ROIs centrally in the LM and proximal RCA. ROIs in the proximal coronaries were as large as possible without including the vessel wall. To determine proximal vessel contrast enhancement, the CT attenuation in the connective tissue was measured by placing the ROI immediately next to the vessel contour and subsequently determining the difference in CT attenuation between vessel lumen and the surrounding tissue. SNR was determined by dividing mean attenuation by image noise. CNR was determined by dividing contrast values by image noise [29].

Quantitative evaluation of carotid artery image quality was likewise evaluated. For each Group 1 examination, attenuation was measured for ROIs placed in the bifurcation of the left and right common carotid arteries (CCA), as well as for ROIs in the internal carotid arteries (ICA) at the level of C7. To determine vessel contrast enhancement, the CT attenuation in the sternocleidomastoid muscle (at the same level of the vessel measurements) was measured. Image noise was defined as the standard deviation of attenuation in the sternocleidomastoid muscle. SNR and CNR were measured at the level of the CCA bifurcation. Attenuation values of the internal jugular veins (IJV) at the level of the CCA bifurcations were also measured for the evaluation of contamination. The area of the ROI was kept constant (0.11–0.13 cm^2^) during all measurements.

Measurements of CNR and SNR were performed in a single session. Readers were blinded to the CT protocol used as the images were read in an anonymized fashion. In addition, readers were completely blinded to the clinical -condition of the patients and the indication for examination.

### Radiation Dose Estimates

CT Dose Index (CTDIvol) and dose-length product (DLP) were obtained for all scans using the dose exposure record generated by the scanner console.

### Statistical Analysis

All variables are expressed as mean value ± SD. Statistical analyses were performed using commercially available software (SPSS, version 16.0, Chicago, IL, USA). Differences in patient characteristics, radiation dose, and image quality parameters (image quality, attenuation, contrast enhancement, image noise, SNR, and CNR) were compared using an ANOVA test and Student’s t test for parametric data, Kruskal-Wallis test for non parametric data, as appropriate. The agreement between the two observers in assessing image quality was calculated by means of Cohen’s kappa statistics. Kappa results were interpreted as being either poor (κ < 0.20), fair (κ = 0.21–0.40), moderate (κ = 0.41–0.60), good (κ = 0.61–0.80), very good (κ = 0.81–0.90), or excellent (κ≥0.91). A P-value of less than 0.05 was considered significant.

## Results

In all eligible patients, CTA examinations were carried out successfully. No complications, side effects, or technical failures occurred. Scan times were 1.31±0.06, 0.38±0.01, and 0.42±0.02 seconds and scan range were 504±28, 133.5±11, and 135.5±12 mm for Group 1, 2 and 3, respectively.

### Patient Characteristics

Patients from Groups 1, 2 and 3 showed no significant differences in age, sex, or BMI, heart rate (Table 1).

**Table 1:** Patient Characteristics for the Three Scanning Protocol Groups.

Characteristics	Group1	Group2	Group3	p
No. of patients	47	51	53	
No. of women	20	23	24	NS
Age (y)	56.6±9.9	62.2±10.2	59.9±10.0	NS
Heart rate (bpm)	58.9±4.9	57.6±5.3	57.0±5.0	NS
BMI (kg/m^2^)	23.6±6.6	24.7±3.4	24.8±2.7	NS

NS = Not significant.

### Comparison of Image Quality

#### Cardiac Assessment

For Group 1, Group 2 and Group 3: mean attenuation of the aortic root was 445.7±68.4, 376.5±50.0, and 402.0±51.8 HU, mean image noise was 29.0±4.7, 23.0±3.5, and 23.9±2.6 HU, and SNR was 15.7±3.5, 16.7±3.2, and 17.0±3.0, respectively. No significant difference in SNR was observed among the three groups.

The image quality of the LM and proximal RCA did not significantly differ between groups (Table 2).

**Table 2:** Comparison of Image Quality and Radiation Dose for Three Scanning Protocol Groups.

Parameter	Group1	Group2	Group3	P
Mean image quality of coronary arteries	1.07±0.31	1.06±0.38	1.04±0.26	0.753
SNR (AO)	15.7±3.5	16.7±3.2	17.0±3.0	0.238
SNR(LM)	15.8±3.2	16.5±3.4	17.3±3.5	0.239
SNR(RCA)	15.6±3.3	16.7±3.2	17.4±3.4	0.100
CNR(LM)	17.9±3.6	19.2±4.3	20.3±4.3	0.073
CNR(RCA)	18.5±3.7	20.2±3.7	20.4±3.9	0.084
CTDIvol (mGy)	4.6±0.4	5.2±1.1	19.0±4.9	<0.01
DLP (mGy×cm)	256.3±24.5	93.4±19.9	254.1±69.9	<0.01

Note-Data are mean±SD. NS = Not significant, SNR = Signal-to-noise ratio, CNR = Contrast-to-noise ratio.

**Figure 2:**
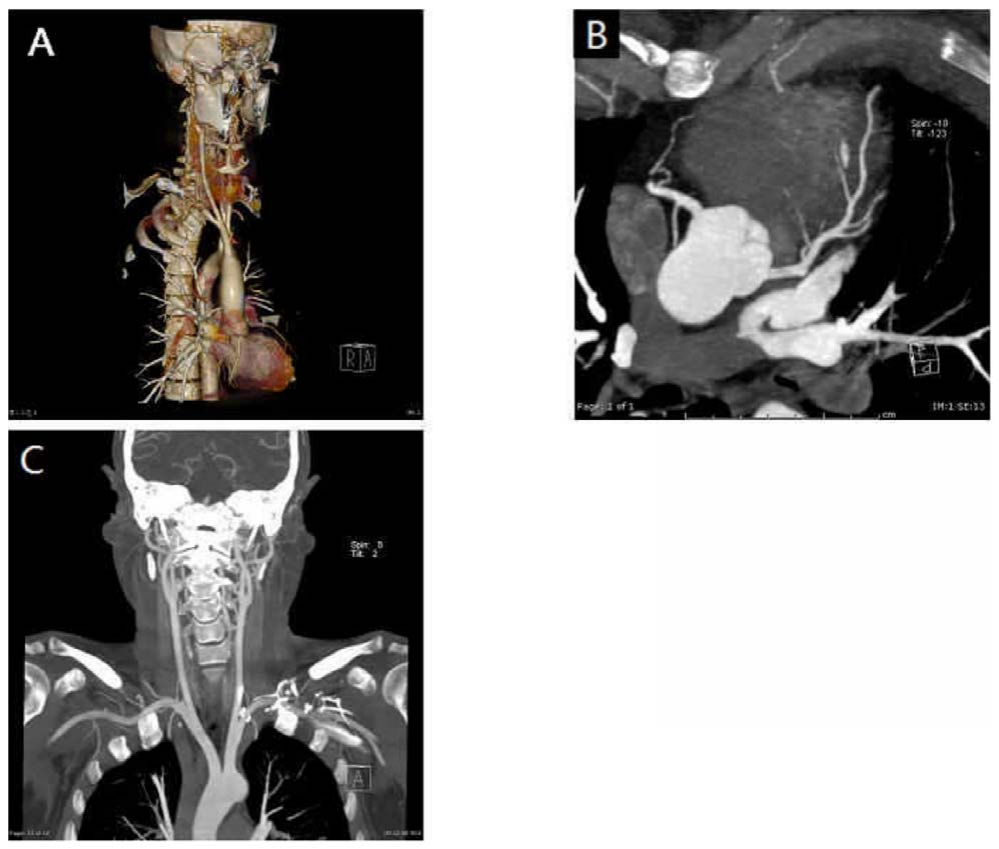
Combined heart, neck and head arteries scan using prospectively ECG- triggered high-pitch spiral scan protocol for a 45-year-old male patient. (a) VRT reconstruction of the whole arteries and MIP reconstructions of the coronary arteries of anterior descending(b) and carotid arteries (c), all with good opacification and definition, without artifacts. DLP was 242 mGy×cm (Scan time: 1.39s; Scan range: 538.5 cm; heart rate: 55 bpm; BMI: 25.4 kg/m^2^).

**Figure 3:**
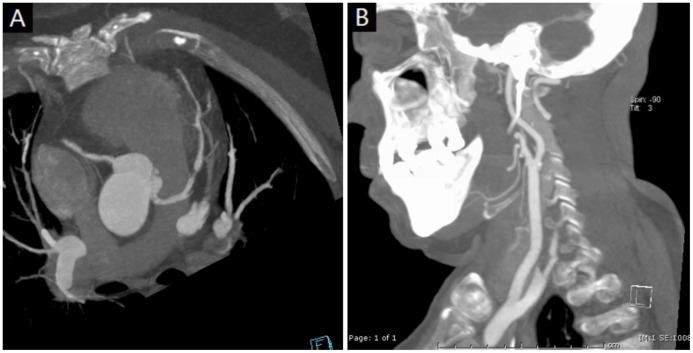
Combined heart, neck and head arteries scan using prospectively ECG- triggered high-pitch spiral scan protocol for a 67-year-old female patient. (a) MIP reconstruction of the coronary arteries revealed significant stenosis of proximal anterior descending and mild stenosis of proximal right coronary artery. (b)MIP reconstruction of left carotid arteries revealed calcification of proximal ICA. DLP was 242 mGy×cm (Scan time: 1.26s; Scan range: 490.0 cm; heart rate: 59 bpm; BMI: 23.1 kg/m^2^).

There were 722 coronary artery segments available for interpretation in Group 1, 789 segments in Group 2, and 824 segments in Group 3 (Figs. 2, 3). Per-patient image quality of the coronary arteries was comparable between Groups: the mean image quality score was 1.07±0.31 in Group 1, 1.06±0.38 in Group 2, and 1.04±0.26 in Group 3 (p = 0.75). No significant difference (p = 0.657) in the number of non-assessable segments was found among Groups: 23/722(3.2%) for Group 1, 23/789(2.9%) for Group 2, and 20/824(2.4%) for Group 1.

The k statistic for interobserver agreement on the image quality for per-coronary-segment analysis was 0.79. Whereas the k statistic for intraobserver agreement was 0.85. The overall interobserver agreement on a per-patient-based analysis was good (κ = 0.73).

#### Image Quality of Carotid Arteries and MCA

In all cases, the carotid arteries were 100% diagnostic (rated good or excellent) in all arterial segments (Fig2, 3). The mean attenuation values at the left and right CCA bifurcation were 542.4±112.5 HU and 531.1±119.3 HU, respectively. The mean attenuation of the C7 segments of the left and right ICA were 462.0±100.2 HU and 444.8±120.5 HU, respectively. The mean SNRs at the level of the left and right CCA bifurcation were 38. 3±16.0 and 38.5±16.8, respectively. The mean CNRs at the level of the left and right CCA bifurcation were 34.1±18.3 and 35.4±17.6, respectively. The mean attenuation values of left and right IJV at the level of the carotid bifurcation were 156.2±84.1HU and 128.7±83.7 HU respectively. The mean image quality of MCA was 1.11±0.31.

### Comparison of Radiation Dose

The mean DLP for Group 1 was 256.3±24.5 mGy•cm, significantly higher than that of Group 2 (93.4±19.9 mGy•cm; p<0.001). However, there was no significant difference of DLP between Group 1 and Group 3 (254.1±69.9 mGy•cm).

## Discussion

This is the first study demonstrating feasibility of high-pitch CTA for the combined evaluation of coronary and carotid arteries. Our results confirm that high-pitch CTA can properly assess in a single examination coronary, epiaortic, and intracranial vessels and demonstrate high diagnostic IQ, excellent interreader variability. Most importantly, this protocol used a low dose of contrast media while delivering a minimal amount of radiation. This protocol could be of significant clinical benefit to patients at risk of concomitant coronary and carotid atherosclerosis.

In the study of Bjerrum[30], more than half of randomly selected apparently healthy middle aged individuals had subclinical atherosclerosis located in the coronary or carotid arteries. Risk stratification of atherosclerosis based on traditional risk factors is limited. The use of noninvasive imaging for direct visualization of atherosclerotic plaques in both coronary and carotid arteries can help to refine risk stratification in certain patients [31]. The comprehensive assessment of coronary and carotid atherosclerosis has also been evaluated using a whole body CTA approach [32], which can be helpful in the detection of multivessel atherosclerosis causing significant vascular stenosis especially in high risk population such as in familial hypercolesterolemia [33]. However, the major drawback have always been high radiation and iodine doses for retrospective ECG-gated coronary CTA with a separate CTA acquisition for extracardiac vasculature. Ultrasound is usually used as the screening modality for atherosclerotic disease of the carotids[34]. However, ultrasound could not detect the lesion of intracranial vessels. Thus, low dose techniques of CTA are necessary for evaluation both coronary and carotid arteries.

The feasibility of the combined cardiac and carotid CTA has been previously investigated in a similar study conducted on patients with suspected stroke [35]. The study demonstrated a good image quality but used a total contrast media dose of 90 mL, significantly higher than our protocol. More importantly, this study employed retrospective-ECG gating for the cardiac acquisition and a CTA of the carotid and intracranial vessels for an average DLP of 1100 mGy•cm—compared with our result of just 256.3 mGy•cm. Prospective electrocardiographically triggered high-pitch spiral technique, which has recently been introduced into clinical practice. In this technique, data acquisition is prospectively triggered by the electrocardiograph and completed within an approximately 270 ms. The radiation dose of combined coronary and extra-coronary high-pitch CTA was comparable to coronary artery-only prospective ECG-triggered sequential scan, corresponding to an radiation exposure of 1.7 times a high-pitch CCTA without head and neck assessment.

The extremely fast acquisition provides excellent intravascular enhancement at a contrast media dose of 65 mL. In particular the mean vascular attenuation fulfilled the requisites for diagnostic studies as previously reported for the coronary [36] and carotid [37]. Moreover, the proper vessel opacification was associated with a good diagnostic quality in all the evaluated regions. In the case of coronary arteries, the overall image quality and number of diagnostic segments was comparable between cardiac high-pitch and sequential-prospective acquisition groups. Regarding carotid arteries, all analyzed segments were also classified as diagnostic and in every case an accurate evaluation of carotid stenosis was possible.

The major limitation in our study is the lack of a direct comparison with a gold standard, such as invasive angiography, for the assessment of diagnostic accuracy in stenosis detection. However, the performance of high-pitch cCTA has been previously established as reliable in CAD detection [14], [18]–[24]. Another limitation is strict heart rate control for high-pitch CTA in this study, and further studies will be required for feasibility of high pitch CTA for the combined evaluation of coronary and carotid arteries in patients with high heart rates.

## Conclusion

The combined heart, neck, and head artery examination using a low contrast, prospectively ECG-triggered high-pitch CTA resulted in excellent vessel enhancement for high quality images at a low radiation dose. This protocol could be of significant clinical benefit to patients at risk of concomitant coronary and carotid atherosclerosis.

## References

[1] Murray CJ, Lopez AD (1997) Mortality by cause for eight regions of the world: Global Burden of Disease Study. Lancet 349: 1269–1276. 9142060 10.1016/S0140-6736(96)07493-4

[2] Gaziano JM (2001) Global burden of cardiovascular disease. In Braunwald E, Zipes DP, Libby P, editors. Heart disease: A textbook of cardiovascular medicine. 6th edition. Philadelphia, PA: WB Saunders Company. pp. 1–17.

[3] Li AH, Chu YT, Yang LH, Chen KC, Chu SH (2007) More coronary artery stenosis, more cerebral artery stenosis? A simultaneous angiographic study discloses their strong correlation. Heart Vessels 22: 297–302. 17879020 10.1007/s00380-006-0971-8

[4] Coskun U, Yildiz A, Esen OB, Baskurt M, Cakar MA, et al (2009) Relationship between carotid intima-media thickness and coronary angiographic findings: a prospective study. Cardiovasc Ultrasound 59: 1–5. 10.1186/1476-7120-7-59 PMC2809045 20043836

[5] Calvet D, Touzé E, Varenne O, Sablayrolles JL, Weber S, et al (2010) Prevalence of asymptomatic coronary artery disease in ischemic stroke patients: the PRECORIS study. Circulation 121: 1623–1629. 20351236 10.1161/CIRCULATIONAHA.109.906958

[6] Steinvil A, Sadeh B, Arbel Y, Justo D, Belei A, et al (2011) Prevalence and predictors of concomitant carotid and coronary artery atherosclerotic disease. J Am Coll Cardiol 57: 779–783. 21310312 10.1016/j.jacc.2010.09.047

[7] Bartlett ES, Walters TD, Symons SP, Aviv RI, Fox AJ (2008) Classification of carotid stenosis by millimeter CT angiography measures: effects of prevalence and gender. Am J Neuroradiol 29: 1677–1683. 18653685 10.3174/ajnr.A1210 PMC8118802

[8] Puchner S, Popovic M, Wolf F, Reiter M, Lammer J, et al (2009) Multidetector CTA in the quantification of internal carotid artery stenosis: value of different reformation techniques and axial source images compared with selective carotid arteriography. J Endovasc Ther 16: 336–342. 19642783 10.1583/08-2636.1

[9] Miller JM, Rochitte CE, Dewey M, Arbab-Zadeh A, Niinuma H, et al (2008) Diagnostic performance of coronary angiography by 64-row CT. N Engl J Med 359: 2324–2336. 19038879 10.1056/NEJMoa0806576

[10] Moon JH, Park EA, Lee W, Yin YH, Chung JW, et al (2011) The diagnostic accuracy, image quality and radiation dose of 64-slice dual-source CT in daily practice: a single institution's experience.Korean J Radiol. 12: 308–318. 10.3348/kjr.2011.12.3.308 PMC3088848 21603290

[11] Johnson TR, Nikolaou K, Busch S, Leber AW, Becker A, et al (2007) Diagnostic accuracy of dual-source computed tomography in the diagnosis of coronary artery disease. Invest Radiol 42: 684–691. 17984765 10.1097/RLI.0b013e31806907d0

[12] Scheffel H, Alkadhi H, Plass A, Vachenauer R, Desbiolles L, et al (2006) Accuracy of dual-source CT coronary angiography: first experience in a high pre-test probability population without heart rate control. Eur Radiol 16: 2739–2747. 17031451 10.1007/s00330-006-0474-0 PMC1705545

[13] Vanhoenacker PK, Heijenbrok-Kal MH, Van Heste R, Decramer I, Van Hoe LR, et al (2007) Diagnostic performance of multidetector CT angiography for assessment of coronary artery disease: meta-analysis. Radiology 244: 419–428. 17641365 10.1148/radiol.2442061218

[14] Weustink AC, Meijboom WB, Mollet NR, Otsuka M, Pugliese F, et al (2007) Reliable high-speed coronary computed tomography in symptomatic patients. J Am Coll Cardiol 50: 786–794. 17707184 10.1016/j.jacc.2007.04.068

[15] Einstein AJ, Henzlova MJ, Rajagopalan S (2007) Estimating risk of cancer associated with radiation exposure from 64-slice computed tomography coronary angiography. JAMA 298: 317–323. 17635892 10.1001/jama.298.3.317

[16] Ardekani MS, Movahed MR, Movafagh S, Ghahramani N (2005) Contrast-induced nephropathy: a review. Cardiovasc Revasc Med 6: 82–85. 16263365 10.1016/j.carrev.2005.07.004

[17] Tublin ME, Murphy ME, Tessler FN (1998) Current concepts in contrast media-induced nephropathy. Am J Roentgenol 171: 933–939. 9762972 10.2214/ajr.171.4.9762972

[18] Achenbach S, Goroll T, Seltmann M, Pflederer T, Anders K, et al (2011) Detection of coronary artery stenoses by low dose, prospectively ECG-triggered, high-pitch spiral coronary CT angiography. JACC Cardiovasc Imaging 4: 328–337. 21492807 10.1016/j.jcmg.2011.01.012

[19] Sommer WH, Albrecht E, Bamberg F, Schenzle JC, Johnson TR, et al (2010) Feasibility and radiation dose of high-pitch acquisition protocols in patients undergoing dual-source cardiac CT. Am J Roentgenol 195: 1306–1312. 21098188 10.2214/AJR.10.4416

[20] Leschka S, Stolzmann P, Desbiolles L, Baumueller S, Goetti R, et al (2009) Diagnostic accuracy of high-pitch dual source CT for the assessment of coronary stenoses: first experience. Eur Radiol 19: 2896–2903. 19760229 10.1007/s00330-009-1618-9

[21] Ertel D, Lell MM, Harig F, Flohr T, Schmidt B, et al (2009) Cardiac spiral dual-source CT with high pitch: a feasibility study. Eur Radiol 19: 2357–2362. 19565245 10.1007/s00330-009-1503-6

[22] Achenbach S, Marwan M, Ropers D, Schepis T, Pflederer T, et al (2010) Coronary computed tomography angiography with a consistent dose below 1 mSv using prospectively electrocardiogram-triggered high-pitch spiral acquisition. Eur Heart J 31: 340–346. 19897497 10.1093/eurheartj/ehp470

[23] Alkadhi H, Stolzmann P, Desbiolles L, Baumueller S, Goetti R, et al (2010) Low-dose, 128-slice, dual-source CT coronary angiography: accuracy and radiation dose of the high-pitch and the step-and-shoot mode. Heart 96: 933–938. 20538669 10.1136/hrt.2009.189100

[24] Neefjes LA, Dharampal AS, Rossi A, Nieman K, Weustink AC, et al (2011) Image quality and radiation exposure using different low-dose scan protocols in dual-source CT coronary angiography: randomized study. Radiology 261: 779–786. 21969666 10.1148/radiol.11110606

[25] Austen WG, Edwards JE, Frye RL, Gensini GG, Gott VL, et al (1975) A reporting system on patients evaluated for coronary artery disease. Report of the Ad Hoc Committee for Grading of Coronary Artery Disease, Council on Cardiovascular Surgery, American Heart Association. Circulation 51: 5–40. 1116248 10.1161/01.cir.51.4.5

[26] Shuman WP, Branch KR, May JM, Mitsumori LM, Lockhart DW, et al (2008) Prospective versus retrospective ECG gating for 64-detector CT of the coronary arteries: comparison of image quality and patient radiation dose. Radiology 248: 431–437. 18552312 10.1148/radiol.2482072192

[27] Beitzke D, Wolf F, Edelhauser G, Plank C, Schernthaner R, et al (2011) Computed tomography angiography of the carotid arteries at low kV settings: a prospective randomised trial assessing radiation dose and diagnostic confidence. Eur Radiol 21: 2434–2444. 21710265 10.1007/s00330-011-2188-1

[28] Fujikawa A, Tsuchiya K, Imai M, Nitatori T (2010) CT angiography covering both cervical and cerebral arteries using high iodine concentration contrast material with dose reduction on a 16 multidetector-row system. Neuroradiology 52: 291–295. 19834700 10.1007/s00234-009-0611-y

[29] Ferencik M, Nomura CH, Maurovich-Horvat P, Hoffmann U, Pena AJ, et al (2006) Quantitative parameters of image quality in 64-slice computed tomography angiography of the coronary arteries. Eur J Radiol 57: 373–379. 16439091 10.1016/j.ejrad.2005.12.023

[30] Bjerrum IS, Sand NP, Poulsen MK, Nørgaard BL, Sidelmann JJ, et al (2013) Non-invasive assessments reveal that more than half of randomly selected middle-aged individuals have evidence of subclinical atherosclerosis: a DanRisk substudy. Int J Cardiovasc Imaging 29: 301–308. 22763580 10.1007/s10554-012-0091-8

[31] Johnson KM, Dowe DA, Brink JA (2009) Traditional clinical risk assessment tools do not accurately predict coronary atherosclerotic plaque burden: a CT angiography study. Am J Roentgenol 192(1): 235–243. 19098205 10.2214/AJR.08.1056

[32] Napoli A, Catalano C, Francone M, Sciacca V, Carbone I, et al (2009) Imaging coronary and extracoronary atherosclerosis: feasibility and impact of whole-body computed tomography angiography. Eur Radiol 19: 1704–1714. 19277677 10.1007/s00330-009-1342-5

[33] Arca M, Quagliarini F, Pigna G, Catalano C, Napoli A (2011) Severe coronary and extracoronary atherosclerosis in autosomal recessive hypercholesterolemia detected by whole-body computed tomography angiography. Intern Emerg Med 6: 571–573. 21380548 10.1007/s11739-011-0550-6

[34] Nair SB, Malik R, Khattar RS (2012) Carotid intima-media thickness: ultrasound measurement, prognostic value and role in clinical practice. Postgrad Med J 88: 694–699. 22761324 10.1136/postgradmedj-2011-130214

[35] Furtado AD, Adraktas DD, Brasic N, Cheng SC, Ordovas K, et al (2010) The triple rule-out for acute ischemic stroke: imaging the brain, carotid arteries, aorta, and heart. Am J Neuroradiol 31: 1290–1296. 20360341 10.3174/ajnr.A2075 PMC7965468

[36] Johnson TR, Nikolaou K, Wintersperger BJ, Fink C, Rist C, et al (2007) Optimization of contrast material administration for electrocardiogram-gated computed tomographic angiography of the chest. J Comput Assist Tomogr 31: 265–271. 17414765 10.1097/01.rct.0000236421.35761.7a

[37] Kim JJ, Dillon WP, Glastonbury CM, Provenzale JM, Wintermark M (2010) Sixty-four-section multidetector CT-angiography of carotid arteries: a systematic analysis of image quality and artifacts. AJNR Am J Neuroradiol 31: 91–99. 19729539 10.3174/ajnr.A1768 PMC7964077

