# Correction: Feasibility of Low-Dose Contrast Medium High Pitch CT Angiography for the Combined Evaluation of Coronary, Head and Neck arteries

**DOI:** 10.1371/journal.pone.0100025

**Published:** 2014-06-06

**Authors:** The PLOS ONE Staff

Equal contribution should have been assigned. The first two authors, Zhiwei Wang and Yu Chen, should be noted as contributing equally to this work and are both first authors.

There is an error in the corresponding author designation. The correct corresponding authors are: Yining Wang (wangyining@pumch.cn), Huadan Xue (bjdanna95@hotmail.com) and Zhengyu Jin (jin_zhengyu@163.com).
